# Interferon-Regulatory Factors Determine Macrophage Phenotype Polarization

**DOI:** 10.1155/2013/731023

**Published:** 2013-11-28

**Authors:** Roman Günthner, Hans-Joachim Anders

**Affiliations:** Nephrologisches Zentrum, Medizinische Klinik und Poliklinik IV, Klinikum der Universität München, Ziemssenstraße 1, 80336 München, Germany

## Abstract

The mononuclear phagocyte system regulates tissue homeostasis as well as all phases of tissue injury and repair. To do so changing tissue environments alter the phenotype of tissue macrophages to assure their support for sustaining and amplifying their respective surrounding environment. Interferon-regulatory factors are intracellular signaling elements that determine the maturation and gene transcription of leukocytes. Here we discuss how several among the 9 interferon-regulatory factors contribute to macrophage polarization.

## 1. Introduction

During development mononuclear phagocyte progenitors populate most tissues where they differentiate into transcriptionally and functionally diverse phenotypes [1–3]; for example, bone marrow, liver, and lung harbor macrophages with an enormous capacity to clear airborne particles, gut-derived pathogens, or cell nuclei expelled from erythroblasts, respectively [4]. In contrast, skin, kidney, and brain host a dense network of dendritic cells [4, 5]. Upon tissue injury M-CSF drives resident mononuclear phagocyte to proliferate [6] or circulating monocytes recruit to the site of injury. It is the local microenvironment that then determines mononuclear phagocyte polarization to distinct phenotypes, which can vary between disorders or between the different stages of a disease process [7]. Several factors mediate mononuclear phagocyte polarization, as being mostly described by in vitro experiments [7, 8]. However, attempts to translate this simplistic model to disease states in vivo often failed to cover all aspects of heterogeneous and changing tissue environments. For example, ischemia-reperfusion injury induces transient sterile inflammation because dying tissue cells release damage-associated molecular patterns (DAMPs) that polarize macrophages toward a classically activated M1-like phenotype [9, 10]. This process is associated with *NF-*κ*B* and *STAT1* pathway activation [2]. Macrophages apoptosis or their phenotype switches towards alternatively activated, M2-like macrophages that produce *IL-10 *and *TGF-*β*,* induce resolution of inflammation, and enforce tissue regeneration [11–15]. Failure of this phenotype switch leads to persistent tissue inflammation, atrophy, and fibrosis [16]. The uptake of neutrophils, epithelium-derived alarmins, and Th2 cytokines *IL-4* and *IL-13* supports this phenotype switch [11]. As disease processes do not always occur in a serial manner, concomitant proinflammatory and anti-inflammatory macrophages infiltrates often populate organs affected by persistent injury, for example, in slowly progressive lesions of organ transplants [17, 18].

Current data suggest that the family of the interferon-regulatory factors (IRFs) plays an important role in regulating macrophage polarization. IRFs are intracellular proteins that regulate immune cell maturation [19]. Here we provide a summary on IRF biology that is focused on the IRF's role in macrophage phenotype control and the associated contributions to tissue inflammation and remodeling.

## 2. The Family of Interferon-Regulatory Factors

**Figure 1:**
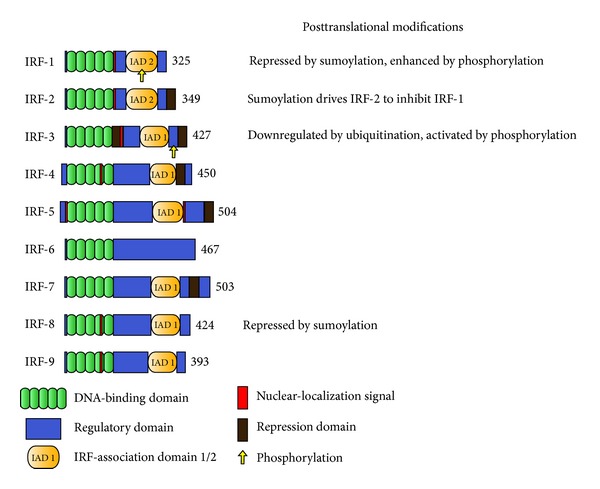
Structural domain organization and important posttranslational modifications of IRFs. Proteins are illustrated by N-terminus on the left and C-terminus on the right. Each of the nine IRFs consists of a conserved pentad repeat DNA-binding domain. Regulatory and repression domains are mostly located in the C-terminal domain. IRF-association domains 1/2(IADs) mediate the interaction with other IRF-family members. Yellow arrows indicate the phosphorylation site within the domain. Posttranslational modifications are illustrated in the right column. Numbers of amino acids for each IRF are given next to structural scheme.

The IRFs were discovered in search of transcription factors that bind to the conserved virus response elements within the promoters of *type I IFN* genes [19]. It was found that both *NF-*κ*B* and *IRF-3* activate *IFN-*β** gene transcription while *IFN-*α** gene expression is entirely based on IRFs [19]. The generation of *Irf*-deficient mice led to the discovery of additional regulatory roles of the IRFs for cell growth, for immune cell maturation and activation, and for apoptosis. In mammals the IRF gene family consists of nine members: *IRF-1, IRF-2, IRF-3, IRF-4, IRF-5, IRF-6, IRF-7, IRF-8/ICSBP, *and* IRF-9*. Their respective IRF proteins share significant homologies at the N-terminal 115 amino acids where they share a conserved tryptophan pentad repeat DNA-binding domain [20]. These include a DNA-binding domain of five tryptophan repeats of which three recognize the GAAA and AANNNGAA sequence motifs, that is, the IFN-stimulated response elements [20]. However, the variable domains at the C-terminus determine the functional specificity of the nine IRFs, their potential to interact with each other via IRF-association domains, and their cell type-specific actions [21] (Figure 1). Accordingly, the IRFs have been subdivided into the “interferonic” IRFs (*IRF-2, -3, -7,* and -*9*), the “stress-responsive” IRFs (*IRF-1* and -*5*), the “hematopoietic” IRFs (*IRF-4* and -*8*), and the “morphogenic” *IRF-6* [22]. The genetic and biological characteristics of the IRF family members are listed in Table 1.

## 3. IRFs in Macrophage Polarization

### 3.1. *IRF-1*

*IRF-1* was first described in 1980s as a 325-amino acid-long nonredundant transcription factor for *type I IFNs* upon *TLR3* ligation [23–25]. *IRF-1* is only weakly expressed in resting DCs and macrophages but is induced by *IFN-*γ** up to 8-fold in M1 polarized macrophages [26]. *IRF-1* interacts with MyD88 to migrate into the nucleus where it triggers TLR-mediated expression of proinflammatory genes [27, 28]. Casein kinase II activates *IRF-1* by phosphorylation [29]. The protein complex formed by *IRF-1, NF-*κ*B,* and *Jun* that bind to the *IFN-*β** promotor was named “enhanceosome” [28, 30–32]. Sumoylation represses the transcriptional activity of *IRF-1* [33]. LPS challenge requires *IRF-1* to induce *TLR3*, *TLR6*, and *TLR9* in macrophages [34]. In fact, *Irf1*-deficient macrophages almost entirely lack inducible nitric oxide synthase (*iNOS*) production upon LPS and *IFN-*γ** stimulation [35]. This way, *IRF-1* contributes to the priming of classically activated, M1-like macrophage polarization in inflammatory tissue environments that involve *IFN-*γ**-producing NKT cells or Th1 T cells [36]. At the same time, *IRF-1* suppresses the binding of other transcription factors to the *IL-4* promoter, which inhibits alternative macrophage activation [37]. This process supports host defense against intracellular pathogens but also accounts for M1 macrophage-related immunopathology [35, 36, 38]. The latter is particularly evident in sterile inflammation, for example, in ischemia-reperfusion injury [39, 40].

### 3.2. *IRF-2*

*IRF-2* is 349-amino acid-long and displays considerable sequence homology with *IRF-1* [23]. *IRF-2* competes with *IRF-1* for the same *cis*-acting recognition sequences in gene promoters [41]. Hence, *IRF-2* is a negative regulator of *IRF-1*-mediated *type I IFN* and *Cox-2* induction [23, 31]. *IRF-2* has a more complex role in cytokine regulation as it suppresses LPS-induced *TNF* expression while augmenting LPS-induced *IL-1, IL-6, IL-12*, and *IFN-*γ** secretion [42]. Sumoylation increases *IRF-2'*s ability to inhibit *IRF-1* transcriptional activity [43]. LPS challenge regulates *TLR3*, *TLR4*, and *TLR5* via *IRF-2* in macrophages [34]. *IRF-2* suppresses caspase-1-mediated programmed cell death by interfering with the transcriptional regulation of caspase-1 and by suppressing STAT1/3 signaling [44]. *Irf-2*-deficient mice are highly susceptible to *Listeria monocytogenes* infection, which seems to be related to *IRF-2'*s role in mediating the *IFN-*γ**-induced oxidative burst that kills the pathogen inside intracellular compartments of macrophages [45]. However, this was *iNOS* transcription independent. *IRF-2* rather regulates* iNOS* in a posttranscriptional manner [46]. The net effect of *IRF-2* on sterile inflammation seems to be immunosuppressive as *Irf-2*-deficient mice are more susceptible to lymphocytic choriomeningitis virus infection as well as to ischemia-reperfusion injury-related tissue inflammation while that latter was suppressed in mice that overexpress *IRF-2* [47]. *IRF-2'*s negative regulatory effect on *type I IFN *expression also suppresses inflammatory skin disease involving CD8 T cells [48]. In addition, *IRF-2* is needed for the development of splenic and epidermal CD4+ dendritic cells [49].

### 3.3. *IRF-3*

*IRF-3* was discovered by searching genes with homology sequences with *IRF-1* and *IRF-2* [50]. This 427-amino acid protein shares a number of characteristics with *IRF-7* [51]. Unlike *IRF-7*, that confers MyD88 signaling, *IRF-3* is involved in TRIF-dependent signaling pathways. After binding pathogens, pattern-recognition receptors like *TLR-3, TLR-4,* or *RIG-I* recruit TRIF to trigger an *IRF-3*-mediated induction of *type I IFN*s [52–55]. Additional cytoplasmic DNA recognition receptors use the STING pathway to activate* IRF-3* [56]. The transcriptional activation of the *IFN-*β** gene requires an enhanceosome of 7 additional proteins that create a continuous surface that recognizes the DNA-binding element [57]. Phosphorylation of *TLR3*'s specific tyrosine residues can initiate two distinct signaling pathways. One activates *TBK-1* and the other activates *PI3 kinase *and *Akt* for full phosphorylation and activation of *IRF-3* [58, 59]. Cytoplasmic *IRF-3* is inactive unless phosphoactivation of *IRF-3* triggers unfolding of the autoinhibitory elements and exposes the hydrophobic surface to interaction with *CREBBP* to translocate to the nucleus [60]. By contrast, ubiquitination inactivates *IRF-3* [61]. GM-CSF-primed M1-like macrophages display a diminished *IRF-3* axis and enhanced activation of MyD88. In contrast, M-CSF stimulated macrophages that develop an M2-like phenotype show defective *NF-*κ*B* activation and enhanced TRIF-mediated *IRF-3* induction upon LPS stimulation [62, 63]. Hence, the *IRF-3* axis is rather enabled in M2-like macrophages than in M1-like macrophages. But does *IRF-3* also contribute to the development of an alternatively activated macrophage phenotype? One study transduced *IRF-3* into primary human microglia. Stimulation with *IFN-*γ*/IL-1* suppressed proinflammatory mediators like *IL-6, TNF-*α*,* or *IL-1*β**, whereas anti-inflammatory mediators including *IL-10* were enhanced [64]. Altogether the data suggest that *IRF-3* is associated with anti-inflammatory microenvironments and contributes to the polarization toward a M2 macrophage phenotype. However, *IRF-3* also induces a number of inflammatory cytokines such as *CCL5* and *IFN-*β** [65].

### 3.4. *IRF-4*

*IRF-4*, first described in 1995, is a 450-amino acid-long “hematopoietic” protein with considerable homology with *IRF-1* and *IRF-2* [66]. *IRF-4* contributes to the maturation of multiple myeloid and lymphoid cell types from their lineage-specific progenitors [19, 67]. *IRF-4* competes with *IRF-5* for binding to the adaptor MyD88 that transmits TLR outside-in signaling to *NF-*κ*B* and other proinflammatory transcription factors [27]. As *IRF-5* is needed for signal transduction the competitive action of *IRF-4* for MyD88 binding renders *IRF-4* an endogenous TLR signaling antagonist that can suppress M1 macrophage polarization [68]. *IL-10* induction needs *IRF-4* and *IRF-4* overexpression enhances *IL-4* and *IL-10* secretion [69]. On the contrary, *IRF-4*−/− mice are more sensitive to LPS-induced sepsis and exhibit higher production of proinflammatory cytokines like *TNF* and *IL-6* [70]. *IL-4* induces macrophages to upregulate *IRF-4* and contributes to their M2 polarization [71]. Accordingly, *IRF-4* deficiency leads to decreased expression of M2 marker genes like *Arg1, Ym1,* and *Fizz1* [72]. In fact, Jumonji domain-containing-3 (Jmjd3), a histone 3 Lys27 (3K27) demethylase, regulates the trimethylation at H3K27 of a selected number of genes including *IRF-4*. This mechanism controls *IRF-4* induction and is needed for M2 macrophage polarization, for example, in the host defense during helminth infection [72]. Interestingly, *IL-4*-induced *STAT6* signaling regulates Jmjd3 [73]. Hence, polarization of alternatively activated macrophages through *IL-4 *seems to be mediated via *STAT6-*Jmjd3*-IRF-4 *signaling and reveals an essential role of *IRF-4* in macrophage polarization for helminth control.

### 3.5. *IRF-5*

*IRF-5* is a 504-amino acid-long stress-responsive IRF [22]. *IRF-5* is required for TLR-mediated induction of *IL-6, TNF, IL-12,* and other proinflammatory cytokines [74]. *IRF-5* competes with *IRF-4* for binding to the signaling adapter MyD88 and the downstream subsequent activation of proinflammatory transcription factors [27]. Its capacity to induce inflammatory cytokines and B cell transcription factors implies its role in host defense and autoimmune disorders [75, 76]. This competitive interaction involves *IRF-5* in the polarization into M1 macrophages [68]. In fact, the balance between *IRF-4* and *IRF-5* seems to be a major determinant of M1 versus M2 macrophage polarization. For example, M-CSF induces *IRF-4* in human monocyte-derived macrophages while GM-CSF induces *IRF-5*, which results in two phenotypically different macrophage phenotypes [77]. M1 macrophages express high levels of *IRF-5* where it not only mediates the expression of proinflammatory cytokines but also suppresses the immunoregulatory cytokine *IL-10* [68]. *IRF-5* itself is regulated by the transcriptional corepressor *KAP1/TRIM28* to avoid overshooting secretion of *TNF* and other mediators that induce immunopathology [78]. *KAP1/TRIM28* regulates *IRF-5* by recruiting histone deacetylases and methyltransferases that can silence *IRF-5*-related gene expression [78]. *IRF-5*-mediated polarization of monocytic phagocytes involves the secretion of various *IL-12* family members including *IL-12p35* and *IL-23p19*, which support Th17 T cell immunity, an element of adaptive immunity that contributes to autoimmune disorders [79]. In fact, gain of function mutations in the *IRF-5* gene exists that increases TLR- or NOD-mediated secretion of proinflammatory cytokines [80]. Such variants also predispose to autoimmune diseases like systemic lupus erythematous [81–83], which may be related to these phenomena.

### 3.6. *IRF-6*

*IRF-6* is a so-called “morphogenic” IRF of 467 amino acid length. *IRF-6* has a large structural homology with *IRF-5* but does not seem to share its functional properties or contribute to macrophage biology, which is related to the tissue-specific expression of *IRF-6. IRF-6* mutations rather predispose to cleft lip or palate and other abnormalities of limb, skin, and craniofacial morphogenesis [84, 85].

### 3.7. *IRF-7*

*IRF-7*, together with *IRF-3*, is a 503-amino acid central and nonredundant mediator of viral nucleic acid-induced induction of *IFN-*α** [19, 86, 87]. *IRF-7* drives the differentiation of monocytes to macrophages but a direct role in macrophage polarization has not been reported.

### 3.8. *IRF-8*

*IRF-8*, also known as interferon consensus sequence-binding protein (ICSBP), is a 393-amino acid-long “hematopoietic” IRF [22]. *IRF-8* (like *IRF-4*) has a dominant role in the maturation and differentiation of monocytes and macrophages from their immature progenitors, while it represses neutrophil production [88–90]. *IFN-*γ**and LPS slow down the intrinsic mobility of *IRF8* inside the nucleus to enforce its chromatin interaction for the initiation of transcription [91]. *IFN-*γ**induces *IRF-8* and *IRF-8 *drives to induction of *IFN-*β**, *IL-12p40, IL-12p35, *and *iNOS* upon TLR stimulation, that is, M1 macrophage gene profile [92]. In addition, *IRF-8 *integrates outside-in signaling of Notch receptors and TLRs for the induction of genes that define an M1 macrophage phenotype [93]. *IRF-8 *selectively modulates *TLR4* signaling via *IRAK2*-dependent activation of *MNK1* and *eIF4E*-regulated translation. *IRF-8* itself is regulated by small ubiquitin-like modifiers *(SUMO) 2/3* at the lysine residue 310. *SUMO3*-conjugated *IRF8* cannot induce *IRF-8* target genes [94]. Upon macrophage activation, SUMOylation of *IRF-8* is reduced as the deSUMOylating enzyme, sentrin-specific peptidase 1 *(SENP1)*, inactivates SUMOylation-related *IRF-8* repression. As such *IRF-8* SUMO conjugation/deconjugation represents a previously unrecognized mechanism of macrophage phenotype control.

### 3.9. *IRF-9*

*IRF-9* is a 424-amino acid-long regulator of *type I IFN* signaling. It forms a DNA-binding complex with the *STAT1* homodimer, for example, for the induction of *CXCL10* [95]. A specific role in macrophage polarization has not been reported.

## 4. Summary and Perspective

**Figure 2:**
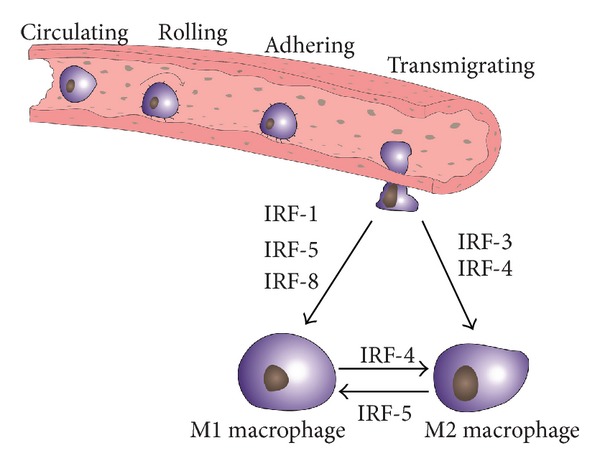
Working model of the role of interferon-regulatory factors in macrophage polarization. Circulating monocytes reach tissues by rolling and adhesion at luminal surfaces of activated endothelia, which is followed by transmigration into the interstitial tissue compartment. The local environment will prime M0 macrophage polarization, a process to which interferon-regulatory factors (IRFs) contribute in a phenotype-specific manner. See text for details.

Macrophages contribute to tissue homeostasis and all phases of tissue injury and repair. Tissue environments prime macrophages to distinct phenotypes to assure that their functional properties enforce the surrounding environment, whether this may be inflammation, the resolution of inflammation, tissue repair (fibrosis), or the resolution of extracellular matrix. Members of the IRF family are an integral component of the macrophage polarization process and, hence, regulate the phenotypic plasticity and heterogeneity of tissue macrophages. Research in this area is still in progress, but our present working model refers to *IRF-1, IRF-5*, and *IRF8* as factors driving the proinflammatory, classically activated (M1) macrophage phenotype, while *IRF-3* and *IRF-4* promote anti-inflammatory, alternatively activated (M2) macrophages (Figure 2). Future work in this area will certainly refine this concept and define additional functions of the IRF's in this context and elucidate additional mechanisms of how changing tissue environments shape immune effector cells to meet the tissue needs in homeostasis and disease.

## Figures and Tables

**Table 1:** Interferon-regulator factors and macrophage polarization.

	Chromosome	Expression	Effect on macrophages	Favoured macrophage phenotype
IRF-1	5q31	Ubiquitous	Induced by IFN-*γ*, mediates TLR/MyD88 signaling, interacts with NF-*κ*B (“enhanceosome”), and suppresses IL-4 promotor	M1

IRF-2	4q34	Ubiquitous	Suppression of IRF-1-mediated IFN and Cox-2 induction, complex role in LPS-induced cytokine release, and suppression of STAT1/3 signalling	Context-dependent

IRF-3	19q13	Ubiquitous	Promotes TRIF-signalling, drives IFN-*γ*/IL-1-mediated IL-10 secretion	M2

IRF-4	6p25	Hematopoietic cells	Induced by IL-4 via Jmjd3, inhibits MyD88 signalling by blocking IRF-5/MyD88 interaction, promotes IL-4 and IL-10 secretion	M2

IRF-5	7q32	Ubiquitous	Interacts with MyD88 needed for MyD88 signalling, drives IL-12p35 and IL-23p19 secretion	M1

IRF-6	1q32	Keratinocytes	—	—

IRF-7	11p15	Ubiquitous	Type I interferon induction	—

IRF-8	16q24	Hematopoietic cells	Induced by IFN-*γ*, mediates TLR-mediated induction of IFN-*β*, IL-12p40, IL-12p35, and iNOS, and mediates Notch and TLR signalling for M1 polarization	M1

IRF-9	14q11	Ubiquitous	Regulates type I interferon signalling	—

TLR: Toll-like receptor, IL: interleukin, IFN: interferon, Cox: cyclooxygenase, LPS: lipopolysaccharide, iNOS: inducible NO synthase.

## Abbreviations

CCL: Chemokine ligand
CCR: Chemokine receptor
Cox-2: Cyclooxygenase 2
CREBBP: CREB-binding protein
DAMP: Danger-associated molecular pattern
eIF4E: Eukaryotic translation initiation factor 4E
GM-CSF: Granulocyte-macrophage colony-stimulating factor
IFN: Interferon
iNOS: Inducible nitric oxide synthase
IRF: Interferon-regulatory factor
Jmjd3: Jumonji domain-containing-3
KAP1: KRAB-associated protein-1
M-CSF: Macrophage colony-stimulating factor
MNK1: MAPK signal-integrating kinase 1
NO: Nitric oxide
NOD: Nucleotide-binding oligomerization domain-containing protein
PAMP: Pathogen-associated molecular pattern
RIG-I: Retinoic acid-inducible gene 1
SENP1: Sentrin-specific protease 1
STAT: Signal transducer and activator of transcription
SUMO: Small ubiquitin-like modifier
STING: Stimulator of interferon genes
TGF-*β*: Transforming growth factor-*β*
TLR: Toll-like receptor
TNF: Tumor necrosis factor
TRIF: TIR-domain-containing adapter-inducing interferon-*β*
TRIM28: Tripartite motif-containing 28.
